# Comparative evaluation of alcoholic handrub: science or marketing?

**DOI:** 10.1186/2047-2994-3-11

**Published:** 2014-04-14

**Authors:** Philippe Berthelot, Martin Exner

**Affiliations:** 1 French Society for Hospital Hygiene (SF2H), Saint-Etienne, France; 2 German Society of Hospital Hygiene and Chairman of the Disinfection, Committee of the German Association for Applied Hygiene, Bonn, Germany

## 

We read with interest but also with some astonishment the paper of Kampf *et al.*[1] comparing the efficacy of three alcoholic-based hand-rubs (ABHR) according to EN 1500 and EN 14476. This study highlighted the possible variability of the results in different laboratories suggesting that the norm methods must be improved for a better standardization (EN 1500 and PrEN 1500). Although the methodology of the experiments seems robust, we do not understand why only three products used in French hospitals were tested and why the positive list of the French Society for Hospital Hygiene (SFHH) was targeted by Kampf *et al.* in the tittle and in the discussion section of the paper. As two of the authors are employed by Bode Chemie GmbH, a competing company of the French companies that manufactured the ABHR tested, there are real competing interests as mentioned at the end of the paper. We want to underscore also that this kind of paper is able to discredit some widely used products, represents a threat to hand hygiene promotion and the confidence in disinfectants if it is misunderstood and misused.

As for some other controversial papers about the efficacy of ABHR [2-4] the French Society for hospital Hygiene and the Disinfectant Commission of the German Association for Applied Hygiene (VAH)^a^ want to remind that independent testing of disinfectants is an important criterion to select active from non- active substances and therefore an essential instrument for quality assurance for disinfectants. To guarantee the activity and reliability under practical conditions the education of healthcare workers in the application of efficient hand disinfectants is a key point. The role of hospital hygiene societies is to promote efficient hand rub use for better hand hygiene in healthcare settings. In 2010, the SFHH choose to end the positive list and begin the writing of guidelines to help healthcare settings to choose disinfectants. The SFHH (now named SF2H) never claimed to be able to control completely the validity of the tests performed and always reminded the readers that the manufacturers and the laboratories that tested the products were responsible for the results of the efficacy tests. On the contrary of Germany, the French Society for Hospital Hygiene do not evaluated on its own responsibility the efficacy of the products. The analyses of the test reports provided by the manufacturer were free and independent. We think that the French positive list has had a positive impact to promote handrub use and despite its limitations permitted to select adequate products for use in healthcare settings [5]. These efforts are necessary also for the future. In Germany the list of tested and by an independent commission of experts approved disinfectants is continued and seemed to be a valuable instrument. In the German philosophy consumer should not be only dependent on the information of companies and industries.

To come back to scientific purpose, this study suggests that the norm methods to test ABHR efficacy must be improved for better standardization, but we don’t agree with all priorities proposed by Kampf *et al.* to improve the validity of products and efficacy claims. We agree with the fulfilling of European pertinent norms and with the necessity to revise regularly the European norms to take into account the observed bias as for example the type of columns in the virucidal test. A better public control of norms and biocide authorizations is needed notably because of the increasing number of news ABHR products.

## Endnote

^a^The Association for Applied Hygiene is an Association of different German Societies for Hygiene including the German Society for Hospital Hygiene.
